# Common variants at 2q11.2, 8q21.3, and 11q13.2 are associated with major mood disorders

**DOI:** 10.1038/s41398-017-0019-0

**Published:** 2017-12-11

**Authors:** Xiao Xiao, Lu Wang, Chuang Wang, Ti-Fei Yuan, Dongsheng Zhou, Fanfan Zheng, Lingyi Li, Maria Grigoroiu-Serbanescu, Masashi Ikeda, Nakao Iwata, Atsushi Takahashi, Yoichiro Kamatani, Michiaki Kubo, Martin Preisig, Zoltán Kutalik, Enrique Castelao, Giorgio Pistis, Najaf Amin, Cornelia M. van Duijn, Andreas J. Forstner, Jana Strohmaier, Julian Hecker, Thomas G. Schulze, Bertram Müller-Myhsok, Andreas Reif, Philip B. Mitchell, Nicholas G. Martin, Peter R. Schofield, Sven Cichon, Markus M. Nöthen, Hong Chang, Xiong-Jian Luo, Yiru Fang, Yong-Gang Yao, Chen Zhang, Marcella Rietschel, Ming Li, Advanced Collaborative Study of Mood Disorder (COSMO) Team, MooDS Bipolar Consortium

**Affiliations:** 1 Key Laboratory of Animal Models and Human Disease Mechanisms of the Chinese Academy of Sciences and Yunnan Province, Kunming Institute of Zoology, Kunming, Yunnan China; 2 Department of Pharmacology, Provincial Key Laboratory of Pathophysiology, Ningbo University School of Medicine, Ningbo, Zhejiang China; 3 Shanghai Key Laboratory of Psychotic Disorders, Shanghai Mental Health Center, Shanghai Jiao Tong University School of Medicine, Shanghai, China; 4 Ningbo Kangning Hospital, Ningbo, Zhejiang China; 5 Brainnetome Center, Institute of Automation, Chinese Academy of Sciences, Beijing, China; 6 Biometric Psychiatric Genetics Research Unit, Alexandru Obregia Clinical Psychiatric Hospital, Bucharest, Romania; 7 Department of Psychiatry, Fujita Health University School of Medicine, Toyoake, Aichi Japan; 8 Laboratory for Statistical Analysis, RIKEN Center for Integrative Medical Sciences, Yokohama, Japan; 9 Laboratory for Omics Informatics, Omics Research Center, National Cerebral and Cardiovascular Center, Osaka, Japan; 10 RIKEN Center for Integrative Medical Sciences, Yokohama, Japan; 11 Department of Psychiatry, Centre Hospitalier Universitaire Vaudois, Prilly, Switzerland; 12 Institute of Social and Preventive Medicine, Centre Hospitalier Universitaire Vaudois, Lausanne, Switzerland; 13 Swiss Institute of Bioinformatics, Lausanne, Switzerland; 14 Department of Epidemiology, Erasmus University Medical Center, Rotterdam, The Netherlands; 15 Institute of Human Genetics, University of Bonn, Bonn, Germany; 16 Department of Genomics, Life and Brain Center, University of Bonn, Bonn, Germany; 17 Human Genomics Research Group, Department of Biomedicine, University of Basel, Basel, Switzerland; 18 Department of Psychiatry (UPK), University of Basel, Basel, Switzerland; 19 Institute of Medical Genetics and Pathology, University Hospital Basel, Basel, Switzerland; 20 Department of Genetic Epidemiology in Psychiatry, Central Institute of Mental Health, Medical Faculty Mannheim, University of Heidelberg, Mannheim, Germany; 21 Institute of Genomic Mathematics, University of Bonn, Bonn, Germany; 22 Institute of Psychiatric Phenomics and Genomics, Ludwig-Maximilians-University Munich, Munich, Germany; 23 Max Planck Institute of Psychiatry, Munich, Germany; 24 Munich Cluster for Systems Neurology (SyNergy), Munich, Germany; 25 University of Liverpool, Institute of Translational Medicine, Liverpool, UK; 26 Department of Psychiatry, Psychosomatic Medicine and Psychotherapy, University Hospital Frankfurt, Frankfurt, Germany; 27 School of Psychiatry, University of New South Wales, Sydney, NSW Australia; 28 Black Dog Institute, Sydney, NSW Australia; 29 QIMR Berghofer Medical Research Institute, Brisbane, QLD Australia; 30 Neuroscience Research Australia, Sydney, NSW Australia; 31 School of Medical Sciences, University of New South Wales, Sydney, NSW Australia; 32 Institute of Neuroscience and Medicine (INM-1), Structural and Functional Organization of the Brain, Genomic Imaging, Research Centre Jülich, Jülich, Germany; 33 Division of Mood Disorders, Shanghai Mental Health Center, Shanghai Jiao Tong University School of Medicine, Shanghai, China; 34 CAS Center for Excellence in Brain Science and Intelligence Technology, Chinese Academy of Sciences, Shanghai, China

## Abstract

Bipolar disorder (BPD) and major depressive disorder (MDD) are primary major mood disorders. Recent studies suggest that they share certain psychopathological features and common risk genes, but unraveling the full genetic architecture underlying the risk of major mood disorders remains an important scientific task. The public genome-wide association study (GWAS) data sets offer the opportunity to examine this topic by utilizing large amounts of combined genetic data, which should ultimately allow a better understanding of the onset and development of these illnesses. Genome-wide meta-analysis was performed by combining two GWAS data sets on BPD and MDD (19,637 cases and 18,083 controls), followed by replication analyses for the loci of interest in independent 12,364 cases and 76,633 controls from additional samples that were not included in the two GWAS data sets. The single-nucleotide polymorphism (SNP) rs10791889 at 11q13.2 was significant in both discovery and replication samples. When combining all samples, this SNP and multiple other SNPs at 2q11.2 (rs717454), 8q21.3 (rs10103191), and 11q13.2 (rs2167457) exhibited genome-wide significant association with major mood disorders. The SNPs in 2q11.2 and 8q21.3 were novel risk SNPs that were not previously reported, and SNPs at 11q13.2 were in high LD with potential BPD risk SNPs implicated in a previous GWAS. The genome-wide significant loci at 2q11.2 and 11q13.2 exhibited strong effects on the mRNA expression of certain nearby genes in cerebellum. In conclusion, we have identified several novel loci associated with major mood disorders, adding further support for shared genetic risk between BPD and MDD. Our study highlights the necessity and importance of mining public data sets to explore risk genes for complex diseases such as mood disorders.

## Introduction

Major mood disorders, including bipolar disorder (BPD) and major depressive disorder (MDD), are leading causes of disability worldwide that account for a substantial proportion of productivity loss, life quality impairment, poor physical health, and deaths by suicide^1,2^. Earlier family and twin studies indicated a pivotal role of genetic factors in the etiology of mood disorders^3–6^; however, the specific modes of inheritance have been found to be complex and polygenic^7–10^. In brief, while BPD and MDD differ from each other in age of onset, clinical presentation, and treatment response^11^, they still share several specific clinical features such as the presence of depressive episodes, mood instability and impaired cognition. Moreover, meta-analyses of family studies have found elevated rates of BPD in first-degree relatives of MDD patients and *vice versa* ^12^. Taken together, there are likely shared genetic components underlying the pathogenesis of BPD and MDD. However, molecular genetic studies have provided limited evidence for this contention so far and only a handful of common risk genes for major mood disorders have been reported to date^13,14^. For example, in a previous study, McMahon et al.^15^ performed a genome-wide association study (GWAS) in a major mood disorder sample of 13,600 individuals, and found a genomic locus at 3p21.1 showing genome-wide significant association, though the risk SNP was latter shown to have stronger association with BPD than with MDD^16,17^. Therefore, understanding the genetic mechanism and pathogenic basis of major mood disorders still remains an important task.

To date, there have been several GWAS conducted in BPD or MDD samples^9,18–38^. Though the current GWAS of mood disorders have identified fewer genome-wide significant genes than expected, their genome-wide statistical results have been (completely or partially) released publicly and these are valuable resources for further larger-scale GWAS meta-analysis. This systematic utilization of these public GWAS resources offers a great opportunity to perform genome-wide screens of the underlying shared genetic factors and will provide valuable information that will benefit other studies of major mood disorders. Therefore, we conducted a genome-wide meta-analysis of GWAS statistics utilizing large samples of individuals diagnosed with BPD or MDD and control subjects, followed by replications of suggestive associations (*p* < 1.0 × 10^–6^) in multiple independent samples from diverse populations (including a total of 32,001 cases and 94,716 controls). This study highlights that there are undiscovered “treasures” underlying the current mood disorder GWAS data sets, and illustrates an example of utilizing available public resources to further dissect the genetic basis of mood disorders.

## Methods

### Research strategy and experimental design

We performed a meta-analysis of two GWAS data sets in a total of 19,637 cases and 18,083 controls of European ancestry. We then evaluated the SNPs with suggestive genome-wide associations (*p* < 1.0 × 10^–6^) in additional 12,364 cases and 76,633 controls from various ethnic groups (Supplementary Table S1). All the protocols and methods used in this study were approved by the institutional review board of the Kunming Institute of Zoology, Chinese Academy of Sciences.

### GWAS data sets meta-analysis

In the discovery stage, we performed a meta-analysis using summary statistics from a BPD GWAS and a non-overlapping MDD GWAS^9,18^. The BPD GWAS sample^9^ comprised 10,410 cases and 10,700 controls that partially overlapped with the Psychiatric Genomics Consortium (PGC1) BPD GWAS sample^20^. Clinical information regarding lifetime history of psychiatric illnesses was collected *via* standardized semi-structured interviews, and lifetime diagnoses were based on operationalized criteria. All cases had experienced pathological episodes of elevated mood (mania or hypomania) and depression and met the criteria for BPD within the primary study classification system. Controls were individuals without BPD selected from the same ethnic groups within the same geographical area. For the MDD analyses, we collected data of 9227 patients and 7383 controls from the PGC1 MDD GWAS sample^18^. The cases were defined by having lifetime diagnoses of MDD according to DSM-IV criteria by trained interviewers, or based on clinician-administered DSM-IV checklists using structured diagnostic instruments. For most of these participants, cases were obtained from clinical sources, and controls were randomly selected from the population. Detailed description of the samples, data quality, genomic controls, and statistical analyses can be found in the original GWAS reports^9,18^.

Illumina OmniExpress, Omni2.5, HumanHap610K, HumanCNV360-Duo or Affymetrix 6.0 were used for the genotyping^9,18^. In both GWAS, strict quality control metrics were applied to ensure the quality of the results. Samples with poor call rates, gender discordance, and/or abnormal heterozygosity as well as those of non-European ancestry were excluded. SNPs with a call rate <95% or Hardy–Weinberg Equilibrium (HWE) *p*-value <1 × 10^–6^ were removed. Each GWAS was imputed separately using IMPUTE2, and SNPs which were poorly imputed (INFO score <0.3) or had minor allele frequency <5% were excluded. There were ~1.3 million SNPs left for the current meta-analysis. In each GWAS, the associations of clinical diagnosis with SNP dosage was tested using logistic regression under an additive model, and covariates such as sample grouping and principal components reflecting the ancestry were taken into consideration. Meta-analyses of GWAS summary statistics were conducted using an inverse variance method under an appropriate effect model according to the test of heterogeneity. Manhattan, quantile–quantile (QQ) and regional plots were made using R (*qqman* package)^39^ and LocusZoom^40^, respectively.

### Replication and technical validation

Replication analyses of the candidate loci were undertaken in two stages in a total of 12,364 cases and 76,633 controls from nine additional samples. The purpose of stage 1 replication was to narrow down the list of candidate SNPs. Since we believe that true genetic risk factors for general populations should show consistent significant associations with mood disorders, we performed this initial replication using the results from either publicly released data sets or from our own samples. After the initial replication, the candidate SNPs underwent the stage 2 replication, in which we collected large samples from international collaborators, and the associations survived stage 1 replication were further tested to confirm their roles in mood disorders. Detailed information of individual samples—including diagnostic assessment, genotyping and quality control—are shown in the Supplemental Data and Supplementary Table S1. The subjects of the replication samples were recruited under relevant ethical and legal guidelines within their respective areas, and all subjects provided written informed consent prior to participation.

There were 40 SNPs reaching the significance threshold of *p* < 1.0 × 10^–6^ in the discovery GWAS meta-analysis and were taken forward for validation in the *Replication sample I*, which included a BPD-type I sample from Romania (Romanian, 451 cases and 318 controls)^41^ and a MDD sample from the CONVERGE consortium (Chinese, 5303 cases and 5337 controls)^21^. These two samples were mainly comprising more homogeneous patients that have been discussed previously^21,41^, ensuring that we can identify true signals using these samples. In addition, we believe that using trans-ethnic sample (CONVERGE) helps to screen for authentic risk signals across populations. The 40 SNPs were first analyzed in each of these two samples and in the total *Replication sample I*, and then meta-analyzed in the total *Replication sample I* combining the discovery GWAS under an inverse variance weighted fixed-effect model. *p*-Values for heterogeneity were calculated using the Cochran’s Q-test. Five SNPs showing *p*-value lower than 5.0 × 10^–8^ in the combined samples were considered genome-wide significant, and were then subject to analyses in *Replication sample II* to further validate the associations.

The data sources of *Replication sample II* were mainly international collaborators, and most of the data sets have been used in previous large-scale studies to identify risk loci for mood disorders^13,14^. In brief, the *Replication sample II* were comprised of 6610 cases and 70,978 controls in total from Australia (330 BPD cases and 1811 controls)^14^, Germany (181 BPD cases and 527 controls)^13^, Japan (2964 BPD cases and 61,887 controls)^22^, GAIN African American (362 BPD cases and 671 controls)^34^, the Netherlands (389 MDD cases and 2056 controls)^38^, Switzerland (the PsyCoLaus cohort of 1301 MDD cases and 1689 controls)^13^, and China (1083 MDD cases and 2337 controls). Association analyses for the risk SNPs were conducted first in each of the above samples alone using logistic regression, and then meta-analyzed in the pooled *Replication sample II* to examine the overall associations. All assays were performed blind to diagnosis and genotype.

### Healthy subjects for expression quantitative trait loci analysis

To identify the impact of risk SNPs on mRNA expression, we utilized two well-characterized gene expression databases to explore the gene expression regulation in the human tissues, providing valuable resources for functional follow-up studies for certain disease-associated variants. We carried out both discovery and replication analyses respectively using each database. We first used the GTEx (Genotype-Tissue Expression project)^42^ data set for the discovery analyses. GTEx contains both genetic variation and RNA-seq gene expression data from a diverse set of human tissues. The dorsolateral prefrontal cortex (DLPFC), hippocampus and cerebellum tissues have been highlighted in the pathogenesis of major mood disorders, we therefore retrieved data of tissues from these brain regions from GTEx for the expression quantitative trait loci (eQTL) analyses. Genes within 200 kb away from each risk SNP were analyzed for eQTL effects of that particular SNP, and Bonferroni correction was applied according to the number of included genes and brain tissues. To replicate the discovery in an independent sample, we conducted the replication analyses using data from the Braineac^43^, the database containing genetic information and whole transcriptome microarray expression information from postmortem brain tissues of 134 normal Caucasians. More detailed information regarding sample collection, processing and analyses can be found in the original studies^42,43^.

## Results

### Discovery GWAS meta-analyses and replication sample I analyses

**Fig. 1:**
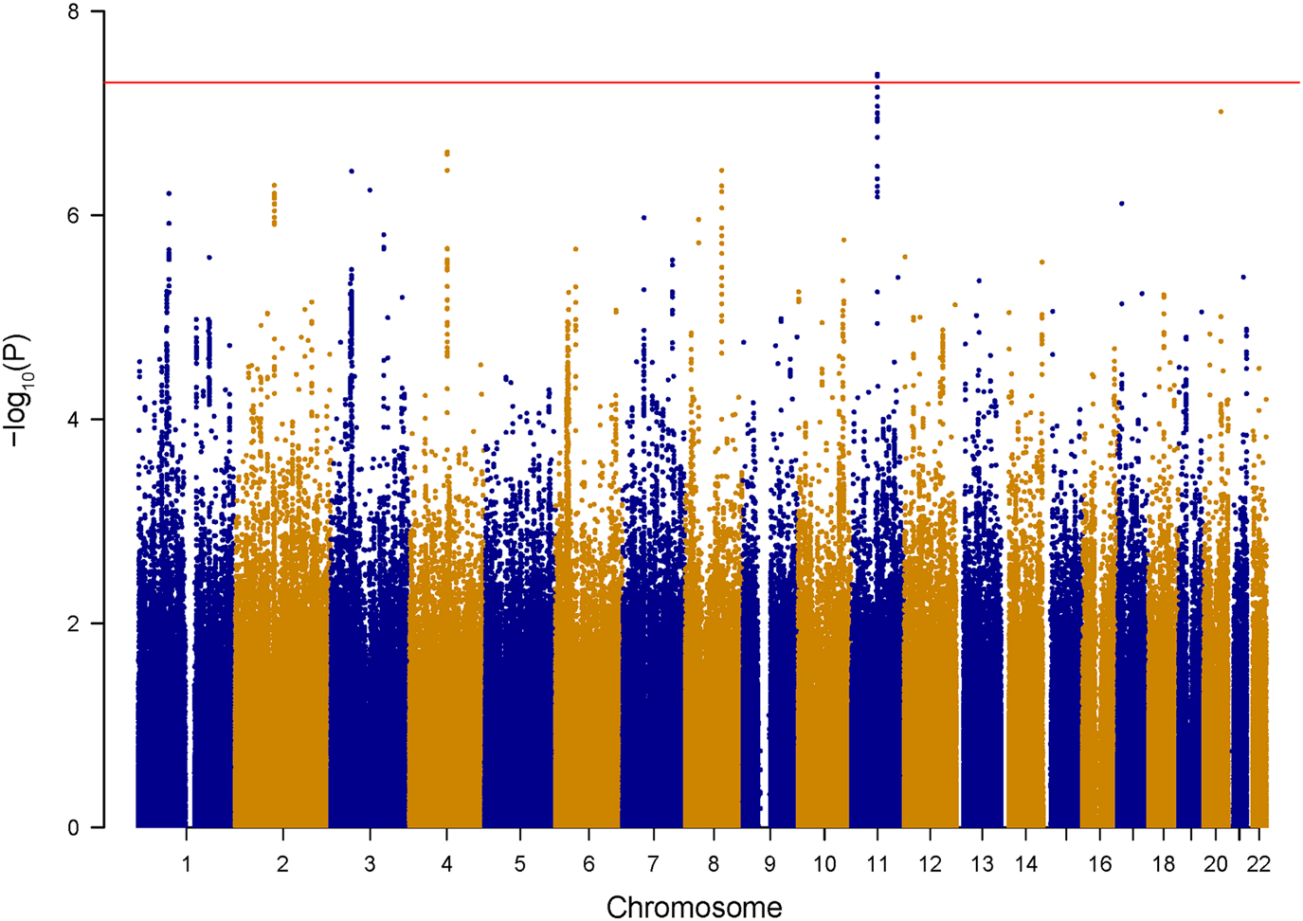
Manhattan plot of the meta-analyses BPD and MDD GWAS data sets Horizontal line indicates threshold for genome-wide significance (*p* < 5 × 10^−8^)

We first conducted a meta-analysis of a BPD GWAS and a MDD GWAS comprising of 19,637 cases and 18,083 controls of European ancestry. After quality control exclusions, ~1.3 million SNPs with minor allele frequency (MAF) > 5% were meta-analyzed in the discovery stage using an appropriate effect model selected according to the heterogeneity test results. We confirmed that the two data sets came from populations with a common distribution by generating the Manhattan and QQ plots (shown in Fig. 1 and Supplementary Fig. S1). The genomic inflation factor (*λ*_GC_) for the meta-analysis was then calculated to be at 1.019. This GWAS meta-analysis yielded a total of nine independent loci (defined by at least 1 Mb separation between each other) reaching suggestive genome-wide significance (*p* < 1.0 × 10^–6^, Supplementary Table S2).

Those suggestive loci were then tested for their associations with major mood disorders in two additional samples (named *Replication sample I* as described above), including 5754 cases and 5655 controls in total, and the majority of the participants in *Replication sample I* were from China (5303 cases and 5337 controls, Supplementary Table S1). Notably, meta-analysis of populations with diverse ethnic backgrounds may increase the power to detect novel complex trait loci. This kind of study exhibits greater resolution for the fine-mapping of causal variants *via* leveraging differences in local linkage disequilibrium (LD) structure between ethnic groups^44^. Therefore, we believe that such trans-ethnic replication (European and Asian populations) will increase our confidence with the authenticity of the identified risk signal. Among the nine independent suggestive loci implicated in the GWAS meta-analysis, we found that three (2q11.2, 8q21.3, and 11q13.2) reached nominal significance in the *Replication sample I* (p < 0.05, Table 1 and S2). Meta-analysis in the sample pool combining discovery GWAS data sets and *Replication sample I* (including a total of 25,391 cases and 23,738 controls) showed that SNPs at 2q11.2, 8q21.3 and 11q13.2 were genome-wide significantly associated with major mood disorders (*p* < 5.0 × 10^–8^, Table 1 and Supplementary Table S2).

**Table 1:** GWAS, replication study, and meta-analysis results for selected SNPs

				rs17022433		rs717454		rs10103191		rs10791889		rs2167457	
CHR				chr2		chr2		chr8		chr11		chr11	
Position				99240090		99389204		92971608		66250401		66398972	
Allele				A/T		T/C		A/G		T/C		T/C	
Frequency				0.406/0.594		0.604/0.396		0.727/0.273		0.205/0.795		0.199/0.801	
		Diagnosis	Case/control	OR (SE)	*P-value*	OR (SE)	*P-value*	OR (SE)	*P-value*	OR (SE)	*P-value*	OR (SE)	*P-value*
GWAS meta-analysis	PGC1	BPD	10,410/10,700	1.093 (0.021)	2.95 × 10^–5^	0.925 (0.021)	2.22 × 10^–4^	1.077 (0.021)	4.24 × 10^–4^	0.890 (0.024)	1.43 × 10^–6^	0.892 (0.024)	1.54 × 10^–6^
	PGC1	MDD	9227/7383	1.069 (0.023)	4.47 × 10^–3^	0.924 (0.024)	7.70 × 10^–4^	1.087 (0.023)	3.04 × 10^–4^	0.943 (0.026)	2.62 × 10^–2^	0.931 (0.026)	6.00 × 10^–3^
	Combined		19,637/18,083	1.082	5.09 × 10^–7^	0.925	6.10 × 10^–7^	1.082	5.16 × 10^–7^	0.914	4.40 × 10^–7^	0.909	6.92 × 10^–8^
Replication sample I	Romania	BPD	451/318	1.265 (0.106)	2.68 × 10^–2^	0.838 (0.106)	9.58 × 10^–2^	1.153 (0.105)	0.176	0.981 (0.118)	0.868	0.994 (0.120)	0.960
	China	MDD	5303/5337	1.058 (0.029)	5.20 × 10^–2^	0.943 (0.029)	4.40 × 10^–2^	1.076 (0.041)	6.70 × 10^–2^	0.909 (0.037)	1.69 × 10^–2^	0.931 (0.038)	8.48 × 10^–2^
	Combined		5754/5655	1.071	1.39 × 10^–2^	0.935	1.60 × 10^–2^	1.086	3.11 × 10^–2^	0.915	1.21 × 10^–2^	0.937	7.05 × 10^–2^
GWAS and replication sample I			25,391/23,738	1.080	2.34 × 10^–8^	0.927	3.23 × 10^–8^	1.082	4.69 × 10^–8^	0.914	1.70 × 10^–8^	0.915	1.69 × 10^–8^
Replication sample II	Australia	BPD	330/1811	1.192 (0.084)	3.73 × 10^–2^	0.812 (0.085)	1.43 × 10^–2^	1.199 (0.085)	3.31 × 10^–2^	0.834 (0.097)	6.03 × 10^–2^	0.843 (0.095)	7.36 × 10^–2^
	Germany II	BPD	181/527	0.998 (0.125)	0.989	0.951 (0.125)	0.686	0.939 (0.124)	0.611	1.117 (0.136)	0.416	1.115 (0.136)	0.423
	Japan	BPD	2964/61,887	1.017 (0.029)	0.561	0.991 (0.029)	0.759	1.031 (0.039)	0.425	0.912 (0.040)	2.14 × 10^–2^	0.923 (0.043)	6.30 × 10^–2^
	GAIN-AA	BPD	362/671	1.076 (0.090)	0.418	0.934 (0.090)	0.448	—	—	—	—	—	—
	Netherlands	MDD	389/2056	1.058 (0.099)	0.409	0.953 (0.101)	0.493	0.977 (0.091)	0.717	1.087 (0.109)	0.269	1.146 (0.116)	8.93 × 10^–2^
	PsyCoLaus	MDD	1301/1689	0.976 (0.054)	0.654	1.006 (0.054)	0.907	1.056 (0.054)	0.316	0.934 (0.061)	0.263	0.952 (0.061)	0.421
	China	MDD	1083/2337	—	—	0.910 (0.054)	8.41 × 10^–2^	—	—	—	—	—	—
	Combined		6610/70,978	1.026	0.249	0.957	7.24 × 10^–2^	1.044	0.117	0.930	1.48 × 10^–2^	0.945	6.79 × 10^–2^
GWAS, replication sample I and II			32,001/94,716	1.065	7.87 × 10^–8^	0.938	2.02 × 10^–8^	1.074	2.55 × 10^–8^	0.918	9.36 × 10^–10^	0.921	4.92 × 10^–9^
Combined BPD samples			14,698/75,914	1.074	1.08 × 10^–5^	0.939	9.62 × 10^–5^	1.072	9.62 × 10^–5^	0.900	8.32 × 10^–8^	0.903	3.06 × 10^–7^
Combined MDD samples			17,303/18,802	1.055	1.52 × 10^–3^	0.937	5.58 × 10^–5^	1.076	7.19 × 10^–5^	0.937	1.00 × 10^–3^	0.939	1.63 × 10^–3^

Although two SNPs in high LD (Europeans, *r* ^*2*^ = 0.97; East Asians, *r* ^*2*^ = 0.99) at 11q13.2 showed genome-wide significant associations with mood disorders in the discovery GWAS meta-analysis (rs7120256, *p* = 4.13 × 10^–8^; rs7119426, *p* = 4.33 × 10^–8^; Fig. 1 and Supplementary Table S2), they were not replicated in the *Replication sample I* (rs7120256, *p* = 0.479; rs7119426, *p* = 0.415; Supplementary Table S2). This phenomenon likely resulted from the genetic heterogeneity of these loci between populations. By contrast, there were other SNPs at 11q13.2 showing suggestive genome-wide significant associations in the GWAS meta-analysis (e.g., rs10791889, *p* = 4.40 × 10^–7^; rs2167457, *p* = 6.92 × 10^–8^; Table 1 and Supplementary Table S2), and were also associated (or marginally associated) with mood disorders in the *Replication sample I* (rs10791889, *p* = 0.012; rs2167457, *p* = 0.071; Table 1 and Supplementary Table S2). In the meta-analysis combining GWAS data sets and *Replication sample I*, we found that the latter two SNPs showed genome-wide significant associations (rs10791889, *p* = 1.70 × 10^–8^; rs2167457, *p* = 1.69 × 10^–8^; Table 1). A detailed LD examination revealed that rs10791889 and rs2167457 were in high LD (Europeans, *r* ^*2*^ = 0.92; Chinese, *r* ^*2*^ = 0.80); both were in strong LD with rs7120256 and rs7119426 in Europeans (*r* ^*2*^ ≥ 0.90), but in much weaker LD in Chinese population (*r* ^*2*^ < 0.60) (Supplementary Fig. S2). These data provide likely explanations for the divergent associations of the SNPs in the *Replication sample I*.

Several SNPs at 2q11.2 and 8q21.3 also achieved genome-wide significance when pooling the GWAS data sets and data of *Replication sample I* together (Supplementary Table S2), with the same direction of allelic effects in both European and Chinese populations. In contrast, other suggestive genome-wide significant SNPs within genomic regions of 1p31.1, 3p21.1, 3q11.2, 4q22.3, 17p12, and 20q13.12 in GWAS meta-analysis of European populations, were not significant in the *Replication sample I* (Supplementary Table S2).

### Validations in replication sample II and joint meta-analyses

**Fig. 2:**
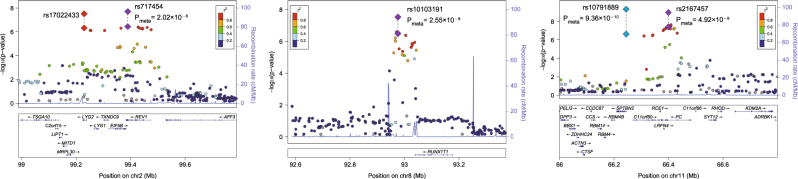
Regional plots of 2q11.2, 8q21.3, and 11q13.2 risk SNPs with major depressive disorder ^40^A physical map of the region is given and depicts known genes within the region, and the European population was used for the construction of LD structure

We further examined the genome-wide significant SNPs at 2q11.2 (rs17022433 and rs717454), 8q21.3 (rs10103191) and 11q13.2 (rs10791889 and rs2167457) in *Replication sample II*, which included a total of 6610 cases and 70,978 controls worldwide. In this replicative analysis, 11q13.2 SNPs were suggestively associated with major mood disorders (rs10791889, *p* = 1.48 × 10^–2^; Table 1), with the same direction of allelic effects as seen in the discovery GWAS meta-analysis. Notably, rs10791889 is the only SNP showing the genome-wide significance in the discovery and *replication* sample *I*, and was also nominally replicated in the independent *replication* sample *II*. The 2q11.2 and 8q21.3 SNPs, though not reaching nominal significance, showed the same trend of association with the discovery analysis (Table 1). We then combined samples from the GWAS datasets plus *Replication sample I* and *II*, which yielded a total of 32,001 cases and 94,716 controls, and conducted joint meta-analyses in this pooled sample. We found that SNPs at 2q11.2 (rs717454), 8q21.3 (rs10103191) and 11q13.2 (rs10791889 and rs2167457) were genome-wide significantly associated with major mood disorders (rs717454, *p* = 2.02 × 10^–8^; rs10103191, *p* = 2.55 × 10^–8^; rs10791889, *p* = 9.36 × 10^–10^; rs2167457, *p* = 4.92 × 10^–9^; Table 1). Regional plots of the risk loci are presented in Fig. 2. SNPs in 2q11.2 and 8q21.3 were novel mood disorders risk SNPs that have not been previously reported, and SNPs at 11q13.2 were in high LD with potential BPD risk SNPs implicated in previous GWAS analyses (rs10896135 was reported in those GWAS with *p*-value of 1.56 × 10^–7^, and it was in high LD with rs10791889 (*r* ^*2*^ > 0.9))^20^. The trans-ethnic replications increased our confidence with the authenticity of the risk loci, and were also consistent with our in prior hypotheses that most of the genome-wide significant SNPs would exhibit similar allelic frequencies between European and Asian populations (Europeans/Asians: rs717454-T, 0.570/0.688; rs10791889-T, 0.267/0.151; rs2167457-T, 0.272/0.133; for the only exception rs10103191, Europeans/Asians: rs10103191-A, 0.481/0.867).

We then stratified the samples based on the case diagnostic status to examine the associations of the genome-wide significant variants with BPD (14,698 cases and 75,914 controls) or MDD (17,303 cases and 18,802 controls) alone. This stratified analysis revealed that the SNPs at 11q13.2 had larger effect sizes and stronger associations with BPD than with MDD, while SNPs at 2q11.2 and 8q21.3 showed similar effect sizes between BPD and MDD (Table 1). All the SNPs were associated with both disorders at nominal significance level.

### Effects of the risk SNPs on nearby gene expression

The association between the risk SNPs with mood disorders in multiple independent samples lends statistical and biological support to the involvement of these genomic regions in risk for these disorders. However, these findings do not identify the underlying molecular mechanisms. Accumulating lines of evidence suggest that genetic risk factors likely contribute to the disease *via* affecting the expression of certain genes^45–48^. To explore whether transcriptional regulation explains the molecular mechanism underlying the risk SNPs identified in our meta-analyses, we carried out investigations using two existing eQTL databases^42,43^.

**Fig. 3:**
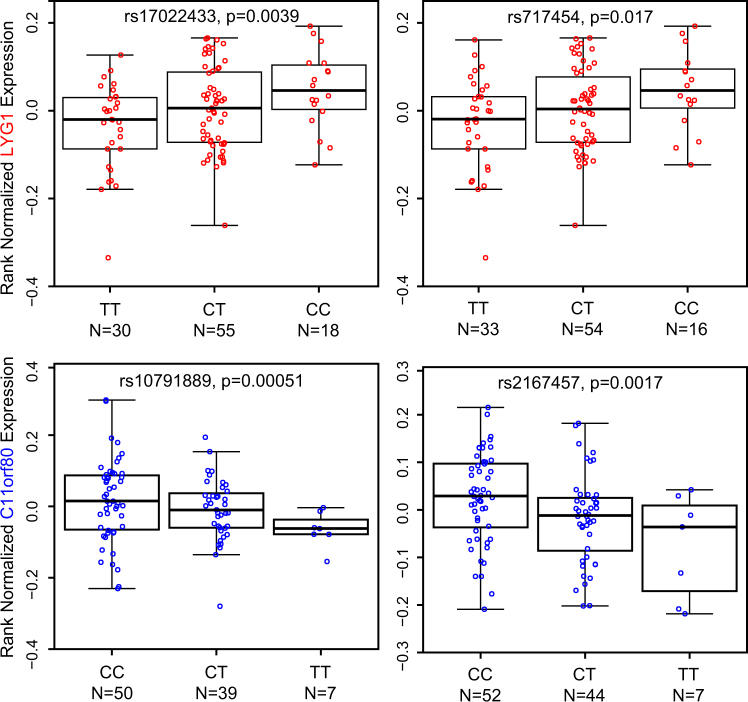
Effects of 2q11.2 and 11q13.2 risk SNPs on nearby gene expression in GTEx data set ^42^

In the GTEx expression database^42^, the 2q11.2 SNPs (rs17022433 and rs717454) were significantly and selectively associated with *LYG1* expression in the cerebellum (rs17022433, *p* = 0.0039; rs717454, *p* = 0.017; Fig. 3). More importantly, such associations in the cerebellum were replicated in the Braineac^43^ (rs17022433, *p* = 0.011; rs717454, *p* = 0.015; Supplementary Fig. S3). Rs10791889 and rs2167457 at 11q13.2 were significantly and specifically associated with the expression of *C11orf80* in the cerebellum in GTEx database (rs10791889, *p* = 0.00051; rs2167457, *p* = 0.0017; Fig. 3). In Braineac, this association was replicated with marginal statistical significance in the cerebellum with the same allelic direction (rs10791889, *p* = 0.055; rs2167457, *p* = 0.079; Supplementary Fig. S3). While *LYG1* and *C11orf80* were the only significant genes in respective genomic risk regions, the eQTL associations survived multiple corrections and were repeatedly observed across independent data sets. Therefore, *LYG1* and *C11orf80* are likely reliable mood disorder related genes, and may contribute to the cerebellum associated pathogenic processes in the disease development. In fact, it has been reported that the cerebellum plays potential roles in psychiatric illnesses *via* its participation in the cortico-ponto-cerebello-thalamo-cortical circuit, by which it communicates with and modulates various congitive domains of the cerebral cortex; additionally, its role in motor coordination and procedural skill learning has been well established^49–51^. Konarski et al.^50^ have initially examined and synthesized the evidence from functional association studies of cerebellar stimulation, lesions, and brain imaging, through which they proposed the landmark hypothesis that abnormalities of the cerebellum play a crucial role in several psychiatric disorders including MDD and BPD.

## Discussion

Major mood disorders are highly heritable traits, but the genetic association (e.g., GWAS) discoveries so far account for only a small portion of the inherited disease risk, which is probably primarily due to the polygenic nature and/or highly heterogeneous genetic architecture of the illnesses. However, GWAS remains an important approach, and it is widely accepted that the accumulation of such discoveries with growing sample sizes will serve as important steps toward the elucidation of biological pathways with etiologic relevance. As a result, mining the current available genetic data sets will help to identify previously undiscovered risk loci, deepening our knowledge of the pathogenesis of major mood disorders and providing valuable information about the genetic basis of these illnesses^52–55^.

The current study is the first report of genome-wide significant association between genetic variants on chromosome 2q11.2 and 8q21.3 and major mood disorders, although a limited number of previous studies have detected suggestive evidence of association in these regions^15,20,31^. We have also confirmed the associations of SNPs on chromosome 11 with mood disorders, which is consistent with a previous BPD GWAS^20^. These lines of evidence suggest potential involvement of those genomic regions in the illnesses, and eQTL analyses in brains have also highlighted certain genes. However, the functions of those genes in brain development and mood disorders are still unclear, so that further investigations are needed.

As previous major mood disorder GWAS have highlighted several genes, located elsewhere in the genome confer the risk of these illnesses^20,30,31,33^, we also tested those genes in our analyses. We detected nominal association for most of them (Supplementary Table S3), but our results cannot be considered as independent replications, as the samples we used partially overlapped with those in the previous studies^20,30,31,33^. A special mention deserves the intergenic SNP rs9834970 (*TRANK1/LBA1* gene) that was not significant in the current meta-analysis of GWAS data sets (Supplementary Table S3) but was significant in the separate analysis of the Romanian BPD-type I sample^41^ and the Japanese BPD sample^22^ with the same direction of the effect as in the discovery study^33^. Notably, the significant results in our study may be different from those of the previous genome-wide analyses^20,30,31,33^, but this is not unexpected since each study has certain limitations in their power to detect the association and other unmeasurable factors such as sampling or technical differences between different studies may also contribute to this inconsistency. Therefore, even with such a large cohort, the aforementioned limitations might affect the power of this meta-analysis to capture all significant risk alleles at the genome-wide level.

Our genetic association results support the previous hypothesis that BPD and MDD share common genetic risk components^12^. However, these data are not sufficient to explain the phenomenon that only certain individuals among the risk variants carriers will develop BPD or MDD, while the others will remain healthy. Potential reasons may include the limited knowledge of the genetic risk variants for these illnesses, environmental influences, epistatic effects between variants, and perhaps unknown epigenetic factors.

This study also highlights the importance and necessity of utilizing public resources to dissect the genetic basis of complex diseases, such as major mood disorders. Many of the published GWAS have released (or partially released) their genome-wide statistical results^18–20^. Even though those GWAS have identified a limited number of genome-wide risk variants due to the sample size, they provided valuable data sets for further analyses and we believe that there are authentic undetected “treasures” underlying these public resources. In this case, we performed a meta-analysis of two public mood disorder GWAS data sets, followed by a set of independent replications, in which some of the replication samples were also available from public sources. Intriguingly, we identified some novel loci for major mood disorders with high confidence, suggesting that this approach is effective in studying major mood disorders, and may also be applicable in investigating other complex diseases.

Despite identifying several risk loci for major mood disorders, this study also raises the concern of potential bias of GWAS. Our current analyses largely rely on data of cases defined with wide diagnostic categories including all possible clinical presentations, and the fact that different cohorts were assembled by different researchers will thus introduce substantial variations regarding the clinical spectrum of cases. For example, systematic variation can occur in potentially important variables including disease symptoms, functional impairment, severity, comorbidity, response to interventions, familial loading, relevant environmental exposures, etc. As a result, the loci conferring risk across a broad phenotypic spectrum are most likely to emerge^56^. Therefore, future studies identifying the loci conferring risk of more specific symptoms are needed^57^, which will require different strategies for phenotypic refinement and the use of multiple large and well-characterized samples. A good example for this idea is the GWAS analyses of female individuals with recurrent MDD to reduce phenotypic heterogeneity, and successfully identified two genome-wide risk loci in a moderate sample size^21^. Besides, there are caveats remain to be addressed in the current study. For example, although we identified novel risk loci for major mood disorders, the lack of experimental investigation prevents us from further understanding their biological roles in the pathogenesis of the illnesses. Future studies on how these SNPs and genes confer risk of mood disorders are thus necessary.

In summary, using public resources, our GWAS meta-analysis followed by independent replications has identified several risk loci for categorically defined BPD and MDD. This is a clear demonstration of the genetic overlap between the major mood disorders that may explain susceptibility to such illnesses and conditions. These results not only contribute to our understanding of the pathogenesis of major mood illnesses, but also provide essential help in future reformation of psychiatric nosology, by contributing to a future scheme reflecting the underlying biology of psychiatric conditions rather than relying solely on the current diagnostic and classification system.

## Electronic supplementary material

SUPPLEMENTAL MATERIAL

Table S1

Table S2

Table S3

Figure S1

Figure S2

Figure S3
