# Supplementary material for: Common variants at 2q11.2, 8q21.3, and 11q13.2 are associated with major mood disorders
Source: Transl Psychiatry. 2017 Dec 11;7:1273. doi: 10.1038/s41398-017-0019-0 (PMC5802692; doi:10.1038/s41398-017-0019-0)
Supplement: Supplementary file 1 — SUPPLEMENTAL MATERIAL [file 41398_2017_19_MOESM1_ESM.pdf]

Additional members from the Bipolar Disorder Group of the MooDS Consortium

Thomas W. Mühleisen[1,2,3,4], Markus Leber[5], Franziska Degenhardt[1,2], Jens Treutlein[6], Manuel Mattheisen[1,7,8], Sandra Meier[6,8,9], Stefan Herms[1,2,4], Per Hoffmann[1,2,3,4], André Lacour[10], Stephanie H. Witt[6], Fabian Streit[6], Susanne Lucae[11], Wolfgang Maier[12], Markus Schwarz[13], Helmut Vedder[13], Jutta Kammerer-Ciernioch[14], Andrea Pfennig[15], Michael Bauer[15], Martin Hautzinger[16], Adam Wright[17,18], Janice M. Fullerton[19,20], Grant W. Montgomery[21], Sarah E. Medland[21], Scott D. Gordon[21], Tim Becker[10,22], Johannes Schumacher[1,2]

1. Institute of Human Genetics, University of Bonn, Bonn, Germany
2. Department of Genomics, Life & Brain Center, University of Bonn, Bonn, Germany
3. Institute of Neuroscience and Medicine (INM-1), Research Centre Jülich, Jülich, Germany
4. Division of Medical Genetics, University Hospital Basel and Department of Biomedicine, University of Basel, Basel, Switzerland
5. Department of Psychiatry & Psychotherapy, University of Cologne, Cologne, Germany
6. Department of Genetic Epidemiology in Psychiatry, Central Institute of Mental Health, Medical Faculty Mannheim, University of Heidelberg, Mannheim, Germany
7. Department of Biomedicine and Centre for integrative Sequencing, iSEQ, Aarhus University, Aarhus, Denmark
8. The Lundbeck Foundation Initiative for integrative Psychiatric Research (iPSYCH), Aarhus and Copenhagen, Denmark
9. National Centre Register-Based Research, Aarhus University, Aarhus, Denmark
10. German Center for Neurodegenerative Diseases (DZNE), Bonn, Germany
11. Max Planck Institute of Psychiatry, Munich, Germany
12. Department of Psychiatry, University of Bonn, Bonn, Germany
13. Psychiatric Center Nordbaden, Wiesloch, Germany
14. Center of Psychiatry Weinsberg, Weinsberg, Germany
15. Department of Psychiatry and Psychotherapy, University Hospital Carl Gustav Carus, TU Dresden, Germany
16. Department of Psychology, Clinical Psychology and Psychotherapy, Eberhard Karls University, Tübingen, Germany
17. School of Psychiatry, University of New South Wales, Sydney, Australia
18. Black Dog Institute, Sydney, New South Wales, Australia
19. Neuroscience Research Australia, Sydney, New South Wales, Australia
20. School of Medical Sciences, University of New South Wales, Sydney, New South Wales, Australia
21. QIMR Berghofer Medical Research Institute, Brisbane, Queensland, Australia
22. Institute for Medical Biometry, Informatics and Epidemiology, University of Bonn, Bonn, Germany

Group Members for the Advanced Collaborative Study of Mood Disorder (COSMO) Team

The advanced COSMO include authors and co-investigators Masashi Ikeda, MD, PhD, Kenji Kondo, MD, PhD, Takeo Saito, MD, PhD, Kunihiro Kawashima, MD, PhD, Yoshio Yamanouchi, MD, PhD, Tsuyoshi Kitajima, MD, PhD, Ayu Shimasaki, MD, Kohei Kawase, MD, Tomohiro Narita, MD, PhD, Taro Kishi, MD, PhD, Kosei Esaki, MD, PhD, Hiroshi Naitoh, MD, PhD, Nakao Iwata (PI), MD, PhD (Department of Psychiatry, Fujita Health University School of Medicine), Tomoko Toyota, MD, PhD, Kazuo Yamada, MD, PhD, Eiji Hattori, MD, PhD, Yoshimi Iwayama, MS, Takeo Yoshikawa (PI), MD, PhD (Laboratory for Molecular Psychiatry, RIKEN Brain Science Institute), Hiroshi Ujike, MD, PhD, Manabu Takaki, MD, PhD, Shinji Sakamoto, MD, Norihito Yamada, MD, PhD, Yuko Okahisa (PI), MD, PhD (Department of Neuropsychiatry, Okayama University Graduate School of Medicine, Dentistry and Pharmaceutical Sciences), Naoki Hashimoto, MD, PhD, Shin Nakagawa, MD, PhD, Yuki Kako, MD, Teruaki Tanaka, MD, PhD, Yutaka Fujii, MD, PhD, Yuji Kitaichi, MD, PhD, Ichiro Kusumi (PI), MD, PhD (Department of Psychiatry, Hokkaido University Graduate School of Medicine), Takeshi Inoue (PI), MD, PhD (Department of Psychiatry, Tokyo Medical University School of Medicine), Kotaro Hattori, MD, PhD, Noriko Yamamoto, Hiroaki Hori, MD, PhD, Toshiya Teraishi, MD, PhD, Miho Ota, MD, PhD, Hiroshi Kunugi (PI), MD, PhD (Department of Mental Disorder Research, National Institute of Neuroscience, National Center of Neurology and Psychiatry), Branko Aleksic, MD, PhD, Itaru Kushima, MD, PhD, Shohko Kunimoto, PhD, Akira Yoshimi, PhD, Toshiya Inada (co-PI), MD, PhD, Norio Ozaki (PI), MD, PhD (Department of Psychiatry, Nagoya University, Graduate School of Medicine), Katsuaki Suzuki, MD, PhD, Yasuhide Iwata, MD, PhD, Norio Mori (PI), MD, PhD (Department of Psychiatry and Neurology, Hamamatsu University School of Medicine), Yoshio Minabe, MD, PhD (Department of Psychiatry and Neurobiology, Kanazawa University Graduate School of Medical Science), Takahiro A. Kato, MD, PhD, Satomi Katsuki, MD, PhD, Hiroshi Mitsuyasu, MD, PhD, Shigenobu Kanba (PI), MD, PhD (Department of Neuropsychiatry, Kyushu University, Graduate School of Medical Sciences), Hiroaki Kawasaki (PI), MD, PhD (Department of Psychiatry, Fukuoka University, Faculty of Medicine), Tsukasa Sasaki (PI), MD, PhD (Laboratory of Health Education, Graduate School of Education, the University of Tokyo), Mamoru Tochigi, MD, PhD (Department of Psychiatry, Teikyo University School of Medicine), Takeshi Otowa, MD, PhD (Department of NeuroPsychiatry, Graduate School of Medicine, the University of Tokyo), Tohru Ohnuma, MD, PhD, Hajime Baba, MD, PhD, Heii Arai (PI), MD, PhD, (Department of Psychiatry, Juntendo University, School of Medicine), Shusuke Numata, MD, PhD, Jun-ichi Iga, MD, PhD, Tetsuro Ohmori (PI), MD, PhD (Department of Psychiatry, Course of Integrated Brain Sciences, Medical Informatics, Institute of Health Biosciences, The University of Tokushima Graduate School), Motohiro Okada, MD, PhD, Yoshiaki Konishi, MS, Hisashi Tanii (PI), MD, PhD, (Department of Psychiatry, Mie University, Graduate School of Medicine), Shigeto Yamawaki, MD, PhD, Yasumasa Okamoto (PI), MD, PhD, (Department of Psychiatry and Neurosciences, Graduate School of Biomedical and Health Sciences, Hiroshima University), Satoshi Saito, MD, (Department of Psychiatry/ Department of Biological Psychiatry and Neuroscience, Dokkyo Medical University School of Medicine), Kazutaka Shimoda, MD, PhD (Department of Psychiatry, Dokkyo Medical University School of Medicine), Kazufumi Akiyama (PI), MD, PhD (Department of Biological Psychiatry and Neuroscience, Dokkyo Medical University School of Medicine), Hirokazu Fujita, MD, PhD, Shinji Shimodera (PI), MD, PhD (Department of Neuropsychiatry, Kochi Medical school, Kochi University), Masatoshi Takeda, MD, PhD, Ryota Hashimoto (PI), MD, PhD (Molecular Research Center for Children's Mental Development, United Graduate School of Child Development, Osaka University; Department of Psychiatry, Osaka University Graduate School of Medicine), Tadafumi Kato (PI), MD, PhD (Laboratory for Molecular Dynamics of Mental Disorders, RIKEN Brain Science Institute), Tetsufumi, Kanazawa, MD, PhD, Hiroshi Yoneda (PI), MD, PhD (Department of Neuropsychiatry, Osaka Medical College), Yuichiro Watanabe, MD, PhD, Toshiyuki Someya (PI), MD, PhD (Department of Psychiatry, Niigata University Graduate School of Medical and Dental Sciences), Reiji Yoshimura (PI), MD, PhD (Department of Psychiatry, University of Occupational and Environmental Health), Masanari Itokawa (PI), MD, PhD (Center for Medical Cooperation, Tokyo Metropolitan Institute of

Medical Science), Masaomi Iyo (PI), MD, PhD (Department of Psychiatry, Chiba University Graduate School of Medicine) and Koji Matsuo (PI), MD, PhD (Division of Neuropsychiatry, Department of Neuroscience, Yamaguchi University Graduate School of Medicine).

Replication Mood Disorder Sample Information

Among all the tested samples, both mood disorder patients and healthy controls provided written informed consent prior to their inclusion in the respective studies. All protocols used in the original studies reporting these samples were approved by the relevant ethical review bodies, and followed all applicable institutional, national and international guidelines.

Romania BPD sample

The Romania sample consisted of 451 BPD patients and 318 healthy controls. All patients were recruited from consecutive hospital admissions and directly interviewed with the Structured Clinical Interview for DSM-IV-TR-Axis I Disorders **-** Patient Version (SCID-I, 1994) and the Diagnostic Interview for Genetic Studies (DIGS) version 3.0 (1999). Information provided by medical records and interviews of family members was also used in a best estimate procedure of diagnosis on the basis of DSM-IV-TR criteria. The control sample was population-based, drawn from the same population as the patients, and was screened for major psychiatric disorders prior to inclusion. The ethnicity of the patients and control subjects was determined by genealogical investigation to the grandparental generation. Only the patient sample was previously reported in other collaborative studies (1-3). The controls were genotyped on Illumina Omni-Express chips at the Life & Brain Center in Bonn, and the patients were also genotyped on Illumina chips (partly on Omni1-Quad).

China MDD sample

The China replication MDD sample was from a published GWAS study by Converge consortium (4). In brief, CONVERGE collected cases of recurrent major depression from 58 provincial mental health centres and psychiatric departments of general medical hospitals in 45 cities and 23 provinces of China. Controls were recruited from patients undergoing minor surgical procedures at general hospitals (37%) or from local community centres (63%). A total of 5,303 Chinese women with recurrent MDD and 5,337 controls without MDD were included in this sample. All subjects were Han Chinese women with four Han Chinese grandparents. Cases were excluded if they had a pre-existing history of bipolar disorder, psychosis or mental retardation. Cases were aged between 30 and 60 and had two or more episodes of MDD meeting DSM-IV criteria with the first episode occurring between 14 and 50 years of age, and had not abused drugs or alcohol before their first depressive episode. All subjects were interviewed using a computerized assessment system. Interviewers were postgraduate medical students, junior psychiatrists or senior nurses, trained by the CONVERGE team for a minimum of 1 week. The diagnosis of MDD was established with the Composite International Diagnostic Interview (CIDI) (WHO lifetime version 2.1; Chinese version), which used DSM-IV criteria. The interview was originally translated into Mandarin by a team of psychiatrists at Shanghai Mental Health Centre, with the translation reviewed and modified by members of the CONVERGE team.

DNA was extracted from saliva samples using the Oragene protocol. A barcoded library was constructed for each sample. All saliva samples were randomized in allocation to sequencing batches, and experimenters performing the sequencing procedure were blinded to sample allocation and outcome assessment. Sequencing reads obtained from Illumina Hi-seq machines were aligned to Genome Reference Consortium Human Build 37 patch release 5 (GRCh37.p5) with Stampy (v1.0.17) using default parameters after filtering out reads containing adaptor sequences or consisting of more than 50% poor quality (base quality #5) bases. Samtools (v0.1.18) was used to index the alignments in BAMformat, and Picardtools (v1.62) was used to mark PCR duplicates for downstream filtering. The Genome Analysis Toolkit's (GATK, version 2.6) BaseRecalibrator was then run on the BAM files to create base quality score recalibration tables, masking known SNPs and INDELs from dbSNP (version 137, excluding all sites added after version 129). Base quality recalibration (BQSR) was then performed on the BAM files using GATKlite (v2.2.15) while also removing read pairs that did not have the 'properly aligned segment' bit set by Stampy (1–5% of reads per sample).

Australia BPD sample

The Australia sample included 330 BPD patients and 1,811 healthy controls. Subjects were ascertained through two studies: 1) a BPD pedigree sample (described in McAuley et al. (5)) and 2) a

specialized Sydney Black Dog Institute BPD clinic sample (described in Mitchell et al. 2009) (6). All subjects were interviewed by trained research staff using the DIGS or SCID, using best-estimate DSM-IV diagnoses derived from those instruments, medical records and FIGS. First, for the pedigree sample, only one BPD subject per family was included in the case sample. Pedigrees were only included in the original genetic study if there was unilineal inheritance, and at least two BPD subjects including at least one with bipolar I disorder. Subjects were ascertained through clinical presentations to the Mood Disorders Unit at the Prince of Wales Hospital in Sydney, direct referrals from Australian clinicians, and BPD consumer organizations. Second, for the clinic sample, subjects comprised consecutive subjects referred by psychiatrists or general practitioners for specialized clinical review. All patients provided written informed consent to participate in this study and the study was approved by the local ethics committee. Patients were included in the MooDS study and genotyped at the Life & Brain Center in Bonn using the Illumina platform.

Australia controls were drawn from families participating in the Brisbane Longitudinal Twin Study, an unselected community sample recruited to take part in studies of melanoma risk factors, cognition, and other phenotypes. Subjects were not screened for any phenotype relevant to BPD. The study was approved by the ethic committee and all probands gave written informed consent. All subjects were genotyped as a single project by deCODE using the Illumina platform and have been through an extensive QC process including exclusion for non-European ancestry. The sample is overwhelmingly of northern European origin, predominately from the British Isles.

Germany II BPD sample

Cases for Germany samples were ascertained from consecutive admissions to the psychiatric inpatient units at the Central Institute for Mental Health in Mannheim, University of Heidelberg, and at the University Hospital Würzburg, as well as at other collaborating psychiatric university hospitals in Germany. DSM-IV lifetime diagnoses of bipolar disorder were assigned using a consensus best-estimate procedure, based on all available information, including semi-structured interviews (AMDP; Germany II), medical records, and the family history method. In addition, the OPCRIT system was used for the detailed polydiagnostic documentation of symptoms (7).

Controls for Germany II were ascertained from the population-based Heinz Nixdorf Recall Study (8). Study protocols were reviewed and approved in advance by Institutional Review Boards of the participating institutions.

All subjects provided written informed consent and were genotyped using the Illumina platform.

Japanese BPD sample

The Japanese BPD sample included two phases sample. The Phase I GWAS initially included 1,612 BPD subjects, with 1,545 BPD cases remaining after genotype quality control (QC); and the Phase II GWAS initially included 1,604 BPD subjects, with 1,419 BPD cases remaining after QC. The diagnosis for each subject followed the DSM-IV-TR criteria for BPD and schizoaffective disorder and was reached by the consensus of at least two experienced psychiatrists, based on unstructured interviews with the subject and their family, as well as a review of the subject's medical records. Subjects were excluded if they had also been diagnosed with an intellectual disability.

As controls, we used GWAS data for subjects in the BioBank Japan project. For the Phase I study, these comprised 7,408 subjects who were genotyped (Illumina HumanOmniExpress v1 chip) in a previous GWAS as case subjects for five non-psychiatric disorders (cerebral aneurysm, esophageal cancer, endometrial cancer, chronic obstructive pulmonary disease and glaucoma) or as healthy volunteers. The controls for the Phase II study included 54,479 subjects who had also been genotyped (Illumina HumanOmniExpressExome v1.2 chip) as case subjects for 14 non-psychiatric disorders: nephrotic syndrome, cancers (stomach, lung, colorectal, prostate and breast), glaucoma, chronic periodontitis, type 2 diabetes (T2D), dyslipidemia, arrhythmia, cerebral infarction, epilepsy and nephrolithiasis, or as healthy controls. The controls were not psychiatrically evaluated. The subjects in Japanese BPD samples were genotyped using the Illumina HumanOmniExpressExome v.1.0/v.1.2 BeadChips.

GAIN-AA BPD sample

Cases were selected from those collected and characterized by the Bipolar Consortium over the past 18 years. All subjects were diagnosed with a standard best estimate (BEFD) procedure. For the BiGS GWA study we selected unrelated Diagnostic and Statistical Manual (DSM) IV-defined BPI subjects. Among those participants, African American (AA) status was based on self-report of at least one grandparent being of AA. A total of 362 of these AA BPD subjects were ultimately included in the BiGS analyses after review of best estimate diagnoses. Controls were ascertained separately through a NIMH-supported contract mechanism between Dr. Pablo Gejman and Knowledge Networks, Inc.; this mechanism allowed the ascertainment of 4,586 subjects across the U.S. who agreed to donate a blood sample for transformation into lymphoblastoid cell lines and to respond to a medical questionnaire. Only individuals with complete or near-complete psychiatric questionnaire data who did not fulfill diagnostic criteria for major depression and denied a history of psychosis or BPD were included as controls for the BiGS analyses. The control groups included 716 AA subjects.

All case subjects were interviewed with the Diagnostic Interview for Genetic Studies. These included diagnosis by DSM-IV, DSM-IIIR, and the Research Diagnostic Criteria (RDC), as well as age of onset, number of episodes for depression, hypomania and mania, temporal relationship of mood disorder to substance abuse and psychosis, evidence of mixed episodes and rapid cycling, and a summary of the family history information. All of these indicators were scored independently by a senior clinician (generally a psychiatrist) based on all available information, including medical records, interviewer observations, the coded DIGS, and the Family Instrument for Genetic Studies ('FIGS,' developed for the NIMH Genetics Initiative; available at http://www.nimhgenetics.org/). The FIGS incorporates clinician judgment on family patterns of illness, including presence or absence of BPD, unipolar disorder, and/or other psychiatric disorders in first and second-degree relatives. Genotyping of the AA samples was carried out using the Affymetrix Genome-Wide Human SNP Array 6.0.

China BPD sample

The patients who met DSM-IV criteria for BPD type 1or type 2 were recruited from the Division of Mood Disorders at Shanghai Mental Health Center, Shanghai Jiao Tong University School of Medicine between November 2006 and October 2010. Each patient was independently interviewed and diagnosed by a consensus of at least two experienced psychiatrists. Diagnoses were further confirmed with an Extensive Clinical Interview and a Structured Clinical Interview for DSM-IV Axis/Disorders, Patient Version (SCID-P) given by a research psychiatrist. Subjects with comorbid diagnosis of other psychiatric disorders or chronic physical illness were excluded in this study to mitigate the potential for compounding factors during our analysis. The Extensive Clinical Interview contains items to assess demographics, mental status, and ages at onset for the BPD patients. To avoid the biases due to the low reliability of retrospective evaluation of prodromal symptoms, we defined age at onset as the first reliably-diagnosed hypo/manic or depressive episode according to DSM-IV criteria.

Control subjects were enrolled from hospital staff and students of the School of Medicine in Shanghai that were interviewed by a specialized psychiatrist with SCID-P. Subjects with any psychiatric disorder and chronic physical disease were excluded from our analysis. All subjects were of Han Chinese origin and provided written informed consent before any study-related procedures were performed. This sample has been reported in a previous study.

PsyCoLaus MDD sample

Subjects were selected from subjects of European ancestry from a community survey (CoLaus) carried out in the city of Lausanne, Switzerland. Subjects were randomly selected from a complete list of the Lausanne inhabitants aged 35-75 years. All 35 to 66-year old participants were invited by letters also to participate in the psychiatric evaluation (PsyCoLaus). Sixty-seven percent of the participants of the CoLaus study in the age range between 35-66 years accepted the psychiatric evaluation, which resulted in a sample of 3,719 individuals, of whom 92% were of European ancestry.

Psychiatric assessment in the PsyCoLaus sub-study included the semi structured Diagnostic Interview for Genetic Studies (DIGS), French version. Cases met DSM-IV criteria for MDD and controls were devoid of any psychiatric disorders. A subset of the 3,419 European subjects who received full psychiatric assessment and gave consent for genetic testing were selected for GWAS genotyping. This research was approved by the local institutional review board. All participants received a detailed description of the goal and funding of the study and signed a written informed consent.

The Netherlands MDD sample

The Netherlands MDD sample is from a recent Erasmus Rucphen Family (ERF) study (9), and includes 389 self-reported or clinically diagnosed patients with MDD and 2,056 healthy controls. The ERF study is a cross-sectional cohort including 3,000 living descendants (age range 18-96 years) of 22 couples who lived in the middle of 18th century in an isolated village in the Southwest of the Netherlands and had at least 6 children baptized in the community church. Until the last few decades descendants of these founders have lived in social isolation with minimal immigration (less than 5%). From the year 1848, the population has expanded from 700 up to 20,000 inhabitants (10). 77% of the fathers and 79% of the mothers in this population have inbreeding coefficient greater than zero. The participants are not selected for any disease or outcome. Detailed information regarding the ERF isolate can be found elsewhere (10-12). The study protocol of the Netherlands was approved by the medical ethics board of the Erasmus MC Rotterdam, the Netherlands. Written informed consents were provided by all the subjects participating in the study.

Chinese MDD sample

In the Chinese MDD sample, we enrolled the MDD samples from the clinical trials: the “OPERATION” (OPtimized trEatment stRAtergies for Treatment-resIstant depressiON) study (13,14) and the “CARE-SSD/MDD” (Construct An Rough Evaluation index system for subsyndromal symptomatic depression and major depressive disorder) study. All patients were diagnosed with MDD strictly according to The Diagnostic and Statistical Manual of Mental Disorders, Fourth Edition (DSM-IV) criteria. Standard diagnostic assessments were supplemented with clinical information obtained by a review of medical records and interviews with family informants. Patients were excluded on the following criteria: (1) those with a lifetime diagnosis of bipolar disorder, schizoaffective disorder, schizophrenia, or another psychotic disorder; as well as (2) female patients who were pregnant, planning to become pregnant, or breast-feeding during the study period. Control subjects were recruited from local volunteers that have no history of mental disorders.

All the patients and control subjects were of Han Chinese origin. This study was performed in accordance with the guidelines laid out in the Declaration of Helsinki as revised in 1989. All subjects were of Han Chinese origin and provided written informed consent before any study-related procedures were performed.

References

1. Vassos E, Steinberg S, Cichon S, Breen G, Sigurdsson E, Andreassen OA, et al. (2012): Replication study and meta-analysis in European samples supports association of the 3p21.1 locus with bipolar disorder. *Biol Psychiatry*. 72:645-50.

2. Hammer C, Cichon S, Muhleisen TW, Haenisch B, Degenhardt F, Mattheisen M, et al. (2012): Replication of functional serotonin receptor type 3A and B variants in bipolar affective disorder: a European multicenter study. *Transl Psychiatry*. 2:e103.

3. Cichon S, Muhleisen TW, Degenhardt FA, Mattheisen M, Miro X, Strohmaier J, et al. (2011): Genome-wide association study identifies genetic variation in neurocan as a susceptibility factor for bipolar disorder. *Am J Hum Genet*. 88:372-81.

4. Converge consortium (2015): Sparse whole-genome sequencing identifies two loci for major depressive disorder. *Nature*. 523:588-91.

5. McAuley EZ, Fullerton JM, Blair IP, Donald JA, Mitchell PB, Schofield PR (2009): Association between the serotonin 2A receptor gene and bipolar affective disorder in an Australian cohort. *Psychiatr Genet*. 19:244-52.

6. Mitchell PB, Johnston AK, Corry J, Ball JR, Malhi GS (2009): Characteristics of bipolar disorder in an Australian specialist outpatient clinic: comparison across large datasets. *Aust N Z J Psychiatry*. 43:109-17.

7. McGuffin P, Farmer A, Harvey I (1991): A polydiagnostic application of operational criteria in studies of psychotic illness. Development and reliability of the OPCRIT system. *Arch Gen Psychiatry*. 48:764-70.

8. Schmermund A, Mohlenkamp S, Stang A, Gronemeyer D, Seibel R, Hirche H, et al. (2002): Assessment of clinically silent atherosclerotic disease and established and novel risk factors for predicting myocardial infarction and cardiac death in healthy middle-aged subjects: rationale and design of the Heinz Nixdorf RECALL Study. Risk Factors, Evaluation of Coronary Calcium and Lifestyle. *Am Heart J*. 144:212-8.

9. Amin N, Belonogova NM, Jovanova O, Brouwer RW, van Rooij JG, van den Hout MC, et al. (2016): Non-synonymous variation in NKPD1 increases depressive symptoms in the European populations. *Biol Psychiatry*.

10. Aulchenko YS, Heutink P, Mackay I, Bertoli-Avella AM, Pullen J, Vaessen N, et al. (2004): Linkage disequilibrium in young genetically isolated Dutch population. *Eur J Hum Genet*. 12:527-34.

11. Lopez-Leon S, Choy WC, Aulchenko YS, Claes SJ, Oostra BA, Mackenbach JP, et al. (2009): Genetic factors influence the clustering of depression among individuals with lower socioeconomic status. *PLoS One*. 4:e5069.

12. Pardo LM, MacKay I, Oostra B, van Duijn CM, Aulchenko YS (2005): The effect of genetic drift in a young genetically isolated population. *Ann Hum Genet*. 69:288-95.

13. Fang Y, Yuan C, Xu Y, Chen J, Wu Z, Cao L, et al. (2011): A pilot study of the efficacy and safety of paroxetine augmented with risperidone, valproate, buspirone, trazodone, or thyroid hormone in adult Chinese patients with treatment-resistant major depression. *J Clin Psychopharmacol*. 31:638-42.

14. Fang Y, Yuan C, Xu Y, Chen J, Wu Z, Cao L, et al. (2010): Comparisons of the efficacy and tolerability of extended-release venlafaxine, mirtazapine, and paroxetine in treatment-resistant depression: a double-blind, randomized pilot study in a Chinese population. *J Clin Psychopharmacol*. 30:357-64.
