# Supplementary material for: Common variants at 2q11.2, 8q21.3, and 11q13.2 are associated with major mood disorders
Source: Transl Psychiatry. 2017 Dec 11;7:1273. doi: 10.1038/s41398-017-0019-0 (PMC5802692; doi:10.1038/s41398-017-0019-0)
Supplement: Supplementary file 2 — Table S1 [file 41398_2017_19_MOESM2_ESM.docx]

**Table S1. Description of individual replication samples included in this study**

| **Sample** | **Diagnosis** | **Ancestry** | **N Cases** | **N Controls** | **Criteria** | **Interview** | **Genotyping** | **References** |
| --- | --- | --- | --- | --- | --- | --- | --- | --- |
| **GWAS meta-analysis** | | | | | | | | |
| PGC1 | BPD | European | 10,410 | 10,700 | DSM-IIR, DSM-IV, RDC | Multiple | Multiple | (9) |
| PGC1 | MDD | European | 9,227 | 7,383 | / | Multiple | Multiple | (18) |
| **Replication sample I** | | | | | | | | |
| Romania | BPD | Romanian | 451 | 318 | DSM-IV | SCID-I-P/DIGS | Illumina | (13) |
| China | MDD | Chinese | 5,303 | 5,337 | DSM-IV | CIDI | Sequencing | (21) |
| **Replication sample II** | | | | | | | | |
| Australia | BPD | Australian | 330 | 1,811 | DSM-IV | SCID,DIGS | Illumina | (13,30) |
| Germany II | BPD | German | 181 | 527 | DSM-IV | AMDP | Illumina | (13,30) |
| Japan | BPD | Japanese | 2,964 | 61,887 | DSM-IV-TR | / | Illumina | (22) |
| GAIN-AA | BPD | African American | 362 | 671 | DSM-IV | DIGS | Affymetrix 6.0 | (34) |
| Netherlands | MDD | Dutch | 389 | 2,056 | DSM-IV | CESD | Illumina | (13,36-38) |
| PsyCoLaus | MDD | Swiss | 1,301 | 1,689 | DSM-IV | DIGS | Illumina | (13) |
| China | MDD | Chinese | 1,083 | 2,337 | DSM-IV | DIGS | SNaPShot | Current Study |

**Abbreviations:**

BPD, bipolar disorder; MDD, major depressive disorder.
