# Supplementary material for: Common variants at 2q11.2, 8q21.3, and 11q13.2 are associated with major mood disorders
Source: Transl Psychiatry. 2017 Dec 11;7:1273. doi: 10.1038/s41398-017-0019-0 (PMC5802692; doi:10.1038/s41398-017-0019-0)
Supplement: Supplementary file 4 — Table S3 [file 41398_2017_19_MOESM4_ESM.docx]

**Table S3. Replication of previous genome-wide significant variants in current GWAS meta-analysis**

| **CHR** | **SNP** | **Position** | **Allele1** | **Allele2** | **Previous studies** | | **Current study** | | **Reference** |
| --- | --- | --- | --- | --- | --- | --- | --- | --- | --- |
|  |  |  |  |  | **P-value** | **OR** | **P-value** | **OR** |  |
| 10 | rs10994397 | 61949130 | T | C | 7.10×10^−9^ | 1.350 | 0.0036 | 1.066 | (20) |
| 6 | rs9371601 | 152832266 | T | G | 4.30×10^−8^ | 1.150 | 2.12×10^−4^ | 1.062 | (20) |
| 11 | rs12576775 | 78754841 | A | G | 4.40×10^−8^ | 0.880 | 0.0068 | 0.944 | (20) |
| 1 | rs4650608 | 79010603 | T | C | 8.35×10^−9^ | / | 6.12×10^−7^ | 1.086 | (20) |
| 2 | rs2271893 | 96769167 | A | G | 2.20×10^−10^ | / | 0.068 | 0.937 | (33) |
| 2 | rs6746896 | 96774676 | A | G | 1.59×10^−8^ | / | 0.072 | 1.065 | (33) |
| 3 | rs7618915 | 52254634 | A | G | 1.64×10^−9^ | / | 0.0018 | 0.949 | (33) |
| 3 | rs9834970 | 36831034 | T | C | 1.48×10^−12^ | / | 0.045 | 0.931 | (33) |
| 10 | rs4948418 | 61855500 | T | C | 3.71×10^−10^ | / | 0.417 | 1.071 | (33) |
| 12 | rs1054442 | 47675587 | C | A | 1.20×10^−8^ | 1.130 | 0.071 | 1.071 | (29) |
| 12 | rs1006737 | 2215556 | A | G | 7.00×10^−8^ | 1.181 | 0.050 | 1.082 | (31) |
| 19 | rs1064395 | 19222735 | A | G | 2.14×10^−9^ | 1.170 | 4.61×10^−5^ | 1.090 | (32) |
| 6 | rs12202969 | 98682944 | A | G | 1.08×10^−8^ | 1.120 | 0.261 | 1.051 | (30) |
