# Supplementary figures and images for: Common variants at 2q11.2, 8q21.3, and 11q13.2 are associated with major mood disorders
Source: Transl Psychiatry. 2017 Dec 11;7:1273. doi: 10.1038/s41398-017-0019-0 (PMC5802692; doi:10.1038/s41398-017-0019-0)

Figure S1. Quantile–quantile plot of GWAS meta-analysis p-values.

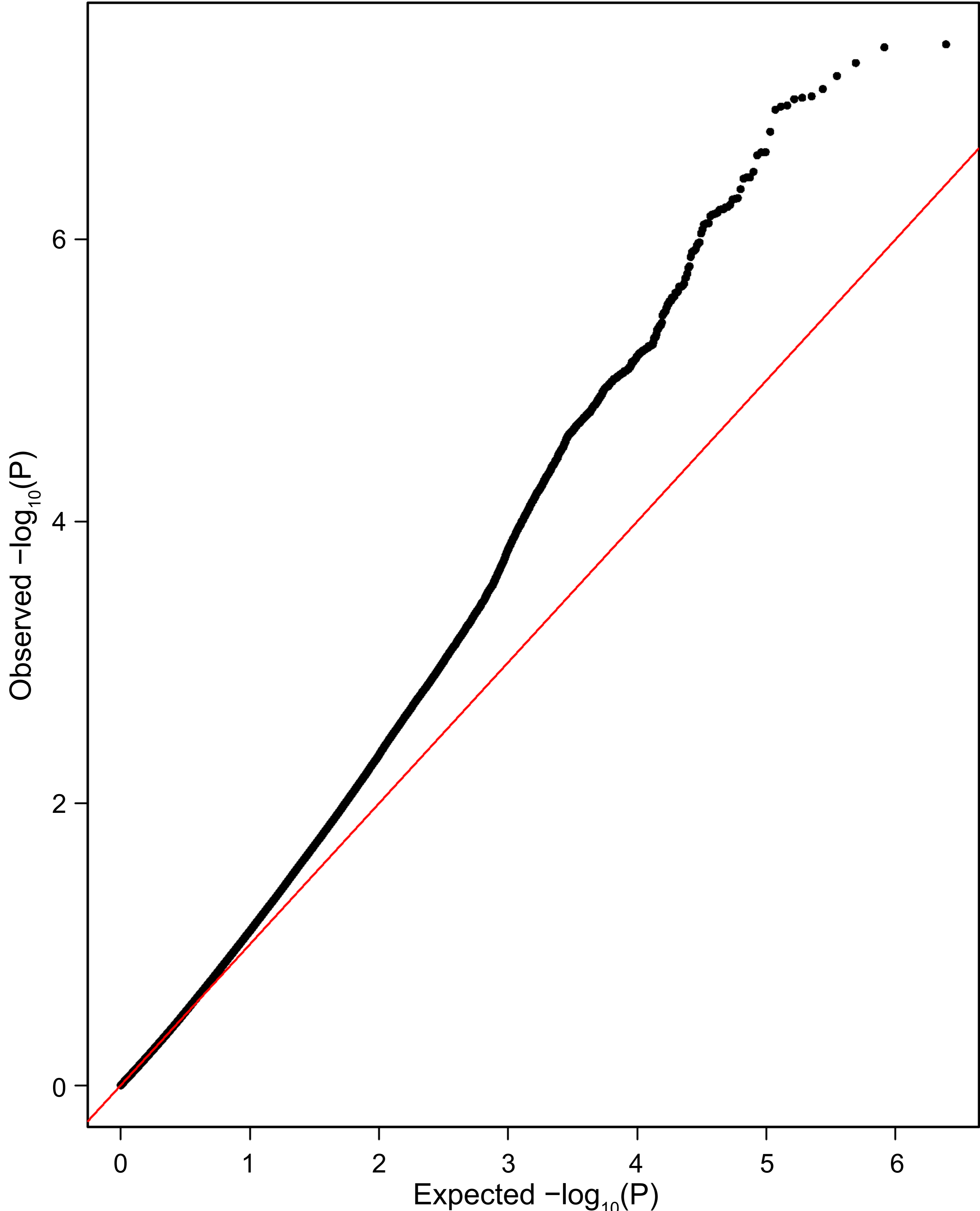

Supplement: Supplementary file 5 — Figure S1 [file 41398_2017_19_MOESM5_ESM.pdf]

Figure S3. Replication of the SNP-Gene eQTL association in Braineac dataset.

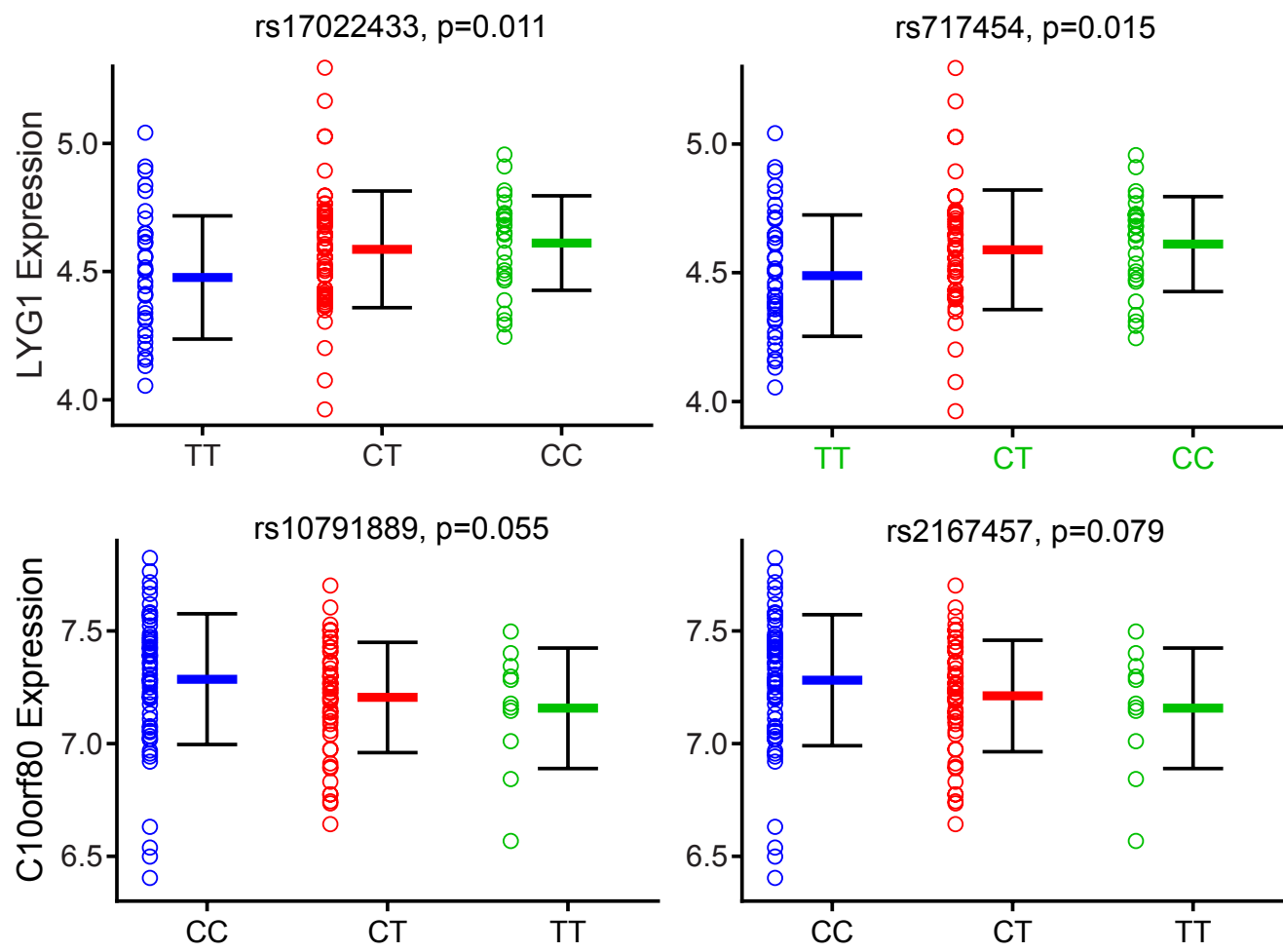

Supplement: Supplementary file 7 — Figure S3 [file 41398_2017_19_MOESM7_ESM.pdf]
