# Supplementary material for: Common variants at 2q11.2, 8q21.3, and 11q13.2 are associated with major mood disorders
Source: Transl Psychiatry. 2017 Dec 11;7:1273. doi: 10.1038/s41398-017-0019-0 (PMC5802692; doi:10.1038/s41398-017-0019-0)

Figure S2. LD patterns between the SNPs at 11q13.2 in European and East Asian populations.

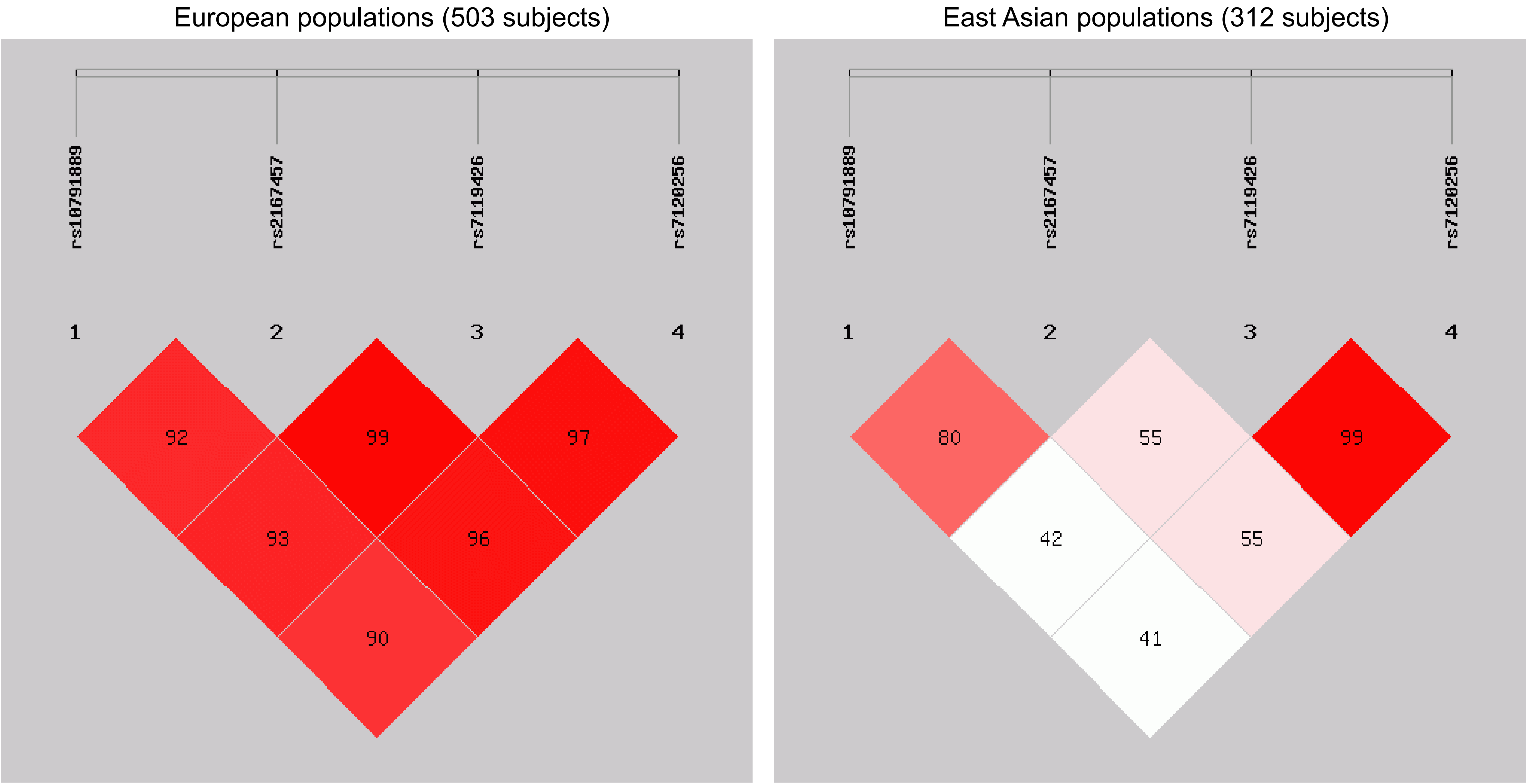

Supplement: Supplementary file 6 — Figure S2 [file 41398_2017_19_MOESM6_ESM.pdf]
